# Humoral Immune Response Induced by the BBIBP-CorV Vaccine (Sinopharm) in Healthcare Workers: A Cohort Study

**DOI:** 10.3390/tropicalmed7050066

**Published:** 2022-04-24

**Authors:** Juan C. Gómez de la Torre, José Alonso Cáceres-DelAguila, Cecilia Muro-Rojo, Nathalia De La Cruz-Escurra, Cesar Copaja-Corzo, Miguel Hueda-Zavaleta, Daniella Arenas Siles, Vicente A Benites-Zapata

**Affiliations:** 1 Clinical Laboratory Roe, Lima 15076, Peru; jgomez@labroe.com (J.C.G.d.l.T.); jcaceres@labroe.com (J.A.C.-D.); cmuro@labroe.com (C.M.-R.); nathalia.delacruz@labroe.com (N.D.L.C.-E.); 2 Faculty of Health Sciences, Universidad Privada de Tacna, Tacna 23003, Peru; cescopajac@upt.pe (C.C.-C.); mighueda@virtual.upt.pe (M.H.-Z.); 3 Hospital III Daniel Alcides Carrión-Essalud Tacna, Tacna 23000, Peru; 4 Faculty of Medicine, Scientific University of the South, Lima 15067, Peru; darenassi@cientifica.edu.pe; 5 Unidad de Investigación para la Generación y Síntesis de Evidencias en Salud, Universidad San Ignacio de Loyola, Lima 15024, Peru

**Keywords:** COVID-19, SARS-CoV-2 vaccination, BBIBP-CorV, healthcare workers, immunoassay, neutralizing antibody

## Abstract

Insufficient data have been reported about the effect of the inactivated SARS-CoV-2 vaccine (BBIBP-CorV) on the humoral response through time in healthcare workers (HCW). This retrospective cohort studied the information of 252 HCW from a private laboratory, comparing the antibody-mediated response provoked by BBIBP-CorV between HCW previously infected with SARS-CoV-2 (PI) and not previously infected (NPI), employing the Elecsys^®^ anti-SARS-CoV-2 S and the cPass™ SARS-CoV-2 Neutralization Antibody Detection kit at intervals of 21, 90, and 180 days after vaccination. The presence of neutralizing antibodies in HCW 21 days after full vaccination was 100% in PI and 91.60% in NPI. We observed a progressive decrease in antibody levels over time in both groups. Comparing HCW PI with NPI, PI had a 10.9, 14.3, and 8.6-fold higher antibody titer with the Elecsys^®^ anti-SARS-CoV-2 S at 21 (*p* < 0.001), 90 (*p*< 0.001) and 180 days (*p* < 0.001) respectively, compared to NPI. Using the percent of signal inhibition (PSI) of the antibody neutralization cPass™, HCW PI showed a level of 1.3, 2.0, and 3.1 times more antibodies, at 21 (*p* < 0.001), 90 (*p* < 0.001), and 180 days (*p* < 0.001) respectively, compared to NPI. We determined a progressive decrease in humoral immunity over time, particularly higher in those NPI.

## 1. Introduction

A diverse set of vaccines have been created against the Severe Acute Respiratory Syndrome Coronavirus 2 (SARS-CoV-2), which have shown to be effective against Coronavirus Disease 2019 (COVID-19) and have been authorized for emergency use [1]. The first vaccine applied to healthcare workers (HCW) in Peru was the BBIBP-CorV (Beijing Bio-Institute of Biological Products Co Ltd., Beijing China), an inactivated SARS-CoV-2 vaccine. This biological product has shown an excellent immunogenicity profile, security, and 78.1% efficacy against SARS-CoV-2 symptomatic disease 14 days after second dose application [2]. Even when the cited clinical trial was not designed to evaluate infection and death prevention efficacy, another study on Peruvian HCW informed 50.4% and 94% efficacy on these results, respectively [3].

Accordant to the immunological protection, a study reported that individuals with positive anti-spike IgG antibodies (Ab) after recovering from COVID-19 presented a reduction in the possibility of reinfections by 88% during a 31-week follow-up [4]. This protection can be compared with other vaccines such as BNT162b2 (Pfizer-BioNTech, New York, USA; Mainz, Germany), mRNA-1273 (Moderna, MA, USA), and ChAdOx1 nCoV-19 (Oxford- AstraZeneca, Oxford, England; Cambridge, England), which have been reported by two months on average after the second dose, protection of 95.0%, 94.1% and 62.1% to 90.0%, respectively [5,6,7]. However, the length of this protective immunity is still unclear. There is a concern over the emergence of SARS-CoV-2 variants, which may affect the COVID-19 vaccines’ efficacy and the acquired protection of previously infected patients [8]

The neutralizing antibodies (NAbs) can prevent viral infection by blocking the entrance of the virus, interfering with the cell surface receptor’s union. These antibodies are employed to estimate vaccine protection against various viral diseases [9]. The presence of NAbs has shown an inverse correlation with the risk of acquiring COVID-19 infection; a 50% inhibitory dilution (ID50) of neutralizing antibody titer of 100 on day 57 increased the vaccine’s efficacy from 50.8% to 90.7% compared to seronegative [10]. In addition, for 50% protection against SARS-CoV-2 infection, a neutralization level of 20.2% of the mean convalescence level is required [11].

Nowadays, the gold standard to evaluate humoral immunity for SARS-CoV-2 is the plaque reduction neutralization test (PRNT) [12]. However (due to its complex process), more practical and standardized tests have been validated, such as the cPass™ SARS-CoV-2 Neutralization Ab detection kit (GenScript, Piscataway, NJ, USA) which demonstrated high sensibility and specificity compared to PRNT [13]. Likewise, the Elecsys^®^ anti-SARS-CoV-2 S test (Roche Diagnostics GmbH, Mannheim, Germany) has shown a high correlation with Ab neutralization cPass™ [14] and moderate correlation compared to PRNT [15]. The Food and Drug Administration (FDA) has approved both tests.

Our study determined the kinetics of neutralizing antibodies in health workers 21, 90, and 180 days after receiving the second dose of the BBIBP-CorV vaccine, and we compared two groups according to previous SARS-CoV-2 infection and seropositivity history.

## 2. Materials and Methods

A retrospective cohort study was designed, analyzing information from a secondary data source obtained from the occupational clinical follow-up of 355 HCW in a private laboratory in Lima, Perú, with or without previous SARS-CoV-2 infection. All the HCW voluntarily agreed to receive two doses of the BBIBP-CoV vaccine, separated by 21 days between doses, and also decided to participate freely in the immune monitoring, sponsored by the laboratory’s occupational health area. The study excluded the HCW with incomplete vaccination, those who did not count with the serological analysis follow-up, and the ones who were infected during the vaccination or post-vaccination period because SARS-CoV-2 infection is responsible for an immune response that could generate bias when comparing titers of antibodies. The study was executed from March 2020 to October 2021.

The study protocol was approved by the medical research ethics committee of the “Universidad Peruana Cayetano Heredia (UPCH)”, the Institutional Research Ethics Committee (CIEI), on 30 September 2021.

Prior to the BBIBP-CorV vaccination, the healthcare workers’ blood samples were evaluated monthly by the laboratory’s occupational health area. This area employed two tests to detect IgM and/or IgG against SARS-CoV-2. One of them was the AESKULISA SARS-CoV-2 S1 for IgG and IgM test (Aesku Diagnostics company, Wendelsheim, Germany), an enzyme-linked immunosorbent assay (ELISA) against the S1 antigen. The other was the Elecsys ^®^ Anti-SARS-CoV-2 (Roche Diagnostics International AG, Rotkreuz, Switzerland), a qualitative electrochemiluminescence immunoassay (ECLIA) that detects antibodies against the SARS-CoV-2 N antigen. The evaluation employing these tests also continued just before the first dose, 21 days after the first dose, and 21 days after the second dose of the BBIBP-CorV vaccine, as a way to establish the immunological fluctuation in HCW.

Post-vaccination, the specific humoral response was analyzed employing the Elecsys^®^ Anti-SARS-CoV-2 S assay and the Ab neutralization cPass™ at three points: (1) 21 days after, (2) 90 days after, and (3) 180 days after the second dose of BBBIBP-CorV.

The Elecsys^®^ Anti-SARS-CoV-2 S is a quantitative ECLIA that detects total high-affinity antibodies (including IgA, IgM, and particularly IgG) against the receptor-binding domain (RBD) of the SARS-CoV-2. The values higher or equal to 0.8 U/mL are considered positive [16]. The cPass™ SARS-CoV-2 Neutralization Ab Detection kit is a qualitative ELISA based on the interaction between the RBD, conjugated with recombinant horseradish peroxidase (HRP) and the human angiotensin-converting enzyme 2 (hACE2), and the sample of the patient. If the patient has NAbs, the interaction between the RBD-ACE2 would be interrupted, and the conjugated HRP would not create a colorimetric signal. The percent of signal inhibition (PSI) was determined by dividing the sample’s optic density (OD) by the OD of the negative control. A PSI equal to or higher than 30% was considered a positive result [17].

During the study period, and as part of the COVID-19 transmission control strategy among HCW, the laboratory’s occupational health area applied molecular tests (qRT-PCR) and a clinical evaluation in all the HCW who reported symptoms or contact with infected individuals. This action was essential to confirm any infected individuals before, during, and after vaccination.

Finally, we separated the HCW secondary data provided by the occupational health area and matched it up into two groups. The first group included all the HWC previously seroconverted (PI) or with a history of SARS-CoV-2 infection. The second group had all the seronegative, without a history of SARS-CoV-2 infection (NPI).

Statistical analysis was performed in STATA V16.0 software (StataCorp., College Station, TX, USA, EE.UU.) and Prism V 9.2.0 (Graphpad Software, LLC, San Diego, CA, USA, EE. UU.). All the numerical variables showed an abnormal distribution and were interpreted using median and interquartile ranges. The categorical variables were analyzed using absolute and relative frequency.

The levels of neutralizing antibodies were compared, between the previous infected and not infected health care personnel, with the Mann–Whitney U test.

The Wilcoxon sign and rank test determined the comparison between NAbs levels during the follow-up in both groups. *p*-values lower than 0.05 (*p* < 0.05) were considered statistically significant.

Employing the crude and adjusted linear regression analysis, we evaluated variables associated with higher results on the Elecsys^®^ anti-SARS-CoV-2 S (U/mL), PSI from the Ab neutralization cPass™. Only those variables that fulfilled the assumptions of linearity, independence of observations, homoscedasticity, and normality of the residuals were included in the analysis.

We evaluated the association between the PSI from the Ab neutralization cPass™ and the levels of the Elecsys^®^ anti-SARS-CoV-2 S using the Spearman correlation coefficient. The results were interpreted according to the correlation’s strength, direction, and statistical significance (*p*-value).

## 3. Results

**Figure 1:**
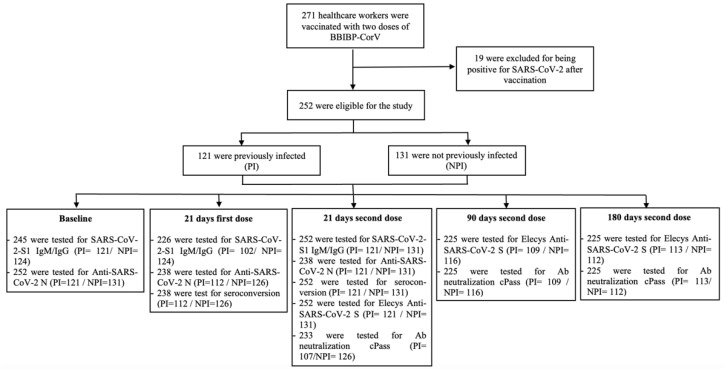
Following healthcare workers after the vaccination and evaluation, according to time. PI: previously infected with SARS-CoV-2, NPI: not previously infected with SARS-CoV-2, Ab: antibodies.

Of the HCW vaccinated with two doses of BBIBP-CorV, 271 were selected for the study (Figure 1). Nineteen HCW were excluded from the study during the follow-up period because they were diagnosed with COVID-19. Finally, we analyzed the results from 252 HCW, 121 (48.01%) previously infected (PI) and 131 (51.99%) not previously infected (NPI) until the vaccination.

In Table 1, the demographic characteristics of the HCW are shown. The median age was 35 years old, and 203 (80.55%) participants were women. 78.35% of the participants worked physically at the laboratories during the pandemic: 30.95% taking samples (phlebotomy), 23.41% in administrative areas, and 20.63% in customer service. They were no statistical differences between the variables from PI and NPI.

### 3.1. Seroconversion

**Figure 2:**
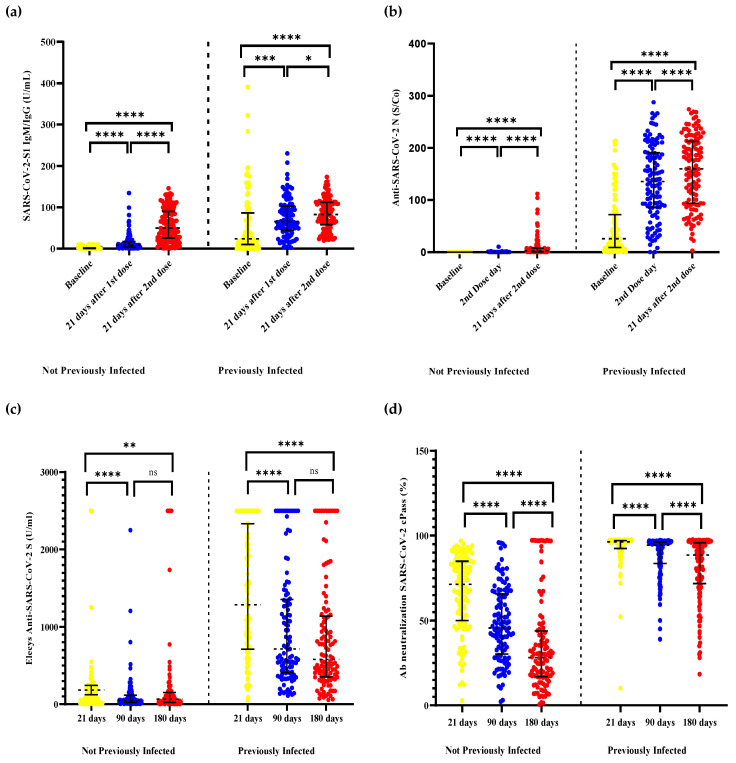
Humoral response kinetics to the inactivated SARS-CoV-2 BBIBP-CorV vaccine, determined up to 180 days after the second dose, between previously infected and not previously infected. (**a**) Effect of the first and second dose BBIBP-CorV on SARS-CoV-2 S1 IgM/IgG; (**b**) Effect of the first and second dose BBIBP-CorV on Anti-SARS-CoV-2 N; (**c**) Titers of total Elecsys^®^ anti-SARS-CoV-2 S after the second dose of BBIBP-CorV vaccination; (**d**) Ab neutralization cPass™ after the second dose of BBIBP-CorV vaccination. ns = no significantly; * = *p* < 0.05; ** = *p* < 0.01; *** = *p* < 0.001; **** = *p* < 0.0001.

The humoral’s kinetic response was determined until 180 days after the second dose of the BBIBP-CorV vaccine (Figure 2). The proportion of seroconversion after 21 days of being vaccinated with the first dose of BBIBP-CorV was 99.10% and 51.59% for PI and NPI, respectively. Twenty-one days after the second dose, the proportion of seroconversion was 100% for PI and 91.60% for NPI. The antibody titration of the SARS-CoV-2 S1 IgM/IgG and anti-SARS-CoV-2 N for evaluating the first and second dose’s effect was higher on PI than NPI (*p* < 0.001). This difference was evident 21 days after receiving the second dose, on which the results of the median SARS-CoV-2 N titers were 55 times higher in the PI group.

### 3.2. Anti-S-RBD IgG by Elecsys^®^ Anti-SARS-CoV-2 S

The proportion of healthcare workers with positive anti-S-RBD IgG at 21 days after the second dose was 100% in PI and 96.94% in NPI (*p* = 0.071). Anti-S-RBD IgG levels in the PI group decreased over time, with median antibody levels of 1288, 714.7, and 558.2 for days 21, 90, and 180, respectively. In the NPI group, a decrease in the level of IgG anti-S-RBD antibodies was also observed on days 21 (median = 117.4) and 90 (median = 49.95); however, an increase in the level of antibodies was observed on day 180 (median = 64.2). Nevertheless, the titers of the PI group were 14 and 8 times higher than those of the NPI group at 90 and 180 days following the second dose of BBIBP-CorV vaccination, respectively (Figure 2).

### 3.3. NAbs against SARS-CoV-2 by Ab Neutralization cPass™

At the 21 days after the second dose of BBIBP-CorV, we observed that the proportion of HCW with positive NAbs against SARS-CoV-2 was 99.06% on PI and 92.06% on NPI (*p* = 0.013). Nonetheless, these positive results were decreasing overtime in the NPI group at the 90th (75% vs. 100%; *p* < 0.001) and 180th day (47.32% vs. 98.23%; *p* < 0.001), compared with the PI group, in which the positive proportion was constantly high (Table 1). The neutralization levels of NAbs decreased over time in both groups. In the HCW PI, we observed a progressive decrease from the 21st (median = 96.4, IQR: 92.4–97.1) to the 90th day (median = 94.3, IQR: 82.9–96.1), and from the 90th to 180th day (median = 88.5, IQR: 72.4–95.8). Nevertheless, even at 180 days, the median NAbs from the PI group were three times higher than the NPI group (Figure 2d, Table 2).

**Figure 3:**
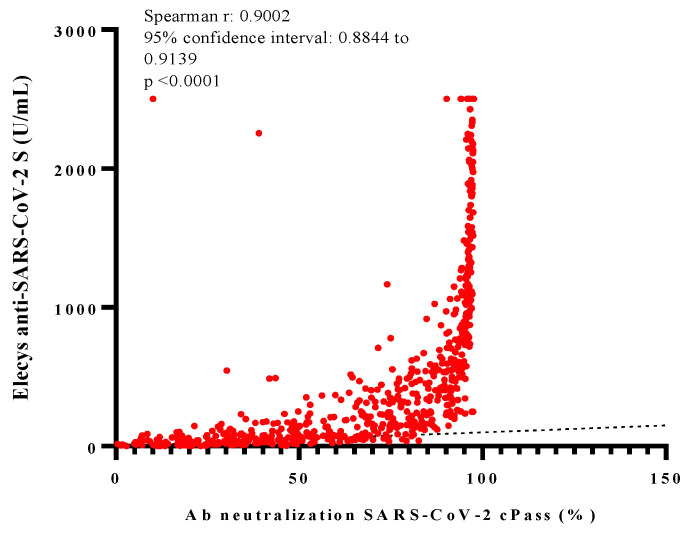
Correlation analysis of values. These graphics show the correlation between antibodies Elecsys^®^ anti-SARS-CoV-2 S and Ab neutralization cPass™. The Spearman correlation efficiency r, 95% confidence interval and *p*-value (two-tailed) are indicated.

When we evaluated the correlation between Elecsys^®^ anti-SARS-CoV-2 S and the Ab neutralization cPass™, we observed a strong positive correlation between both assays (r = 0.900, 95% CI: 0.884 to 0.913; *p* < 0.001) but with an exponential curve (Figure 3).

Finally, employing a multiple linear regression test, we observed that the PI, compared to the NPI, showed a median of 44.69 PSI higher for Ab neutralization cPass™ (*p* < 0.001) and 519.961 UI/mL higher for Elecsys^®^ anti-SARS-CoV-2 S (*p* < 0.001). We further observed that the male patients developed bigger antibody titers on the Elecsys^®^ anti-SARS-CoV-2 S (*p* = 0.006) than females (Table 3).

## 4. Discussion

In this retrospective cohort study of HCW who received two injections of BBIBP-CorV against SARS-CoV-2, we found a robust humoral response after 21 days from the second dose, reaching a 91.6% seroconversion in the NPI group participants. However, this humoral response diminished progressively between the 90th to 180th day after the second dose, particularly in the NPI group, where lower anti-S-RBD IgG and NAbs were detected than those PI. The seroconverted proportion in the study was comparable but discretely lower than the results reported on phases 1 and 2 of the BBIBP-Corv-2 clinical trials [18] and other observational studies [19,20,21], although higher than that reported by Lijeskic [22]. However, we did not find a difference in the proportions of seroconverts according to age group, probably because most of our participants were adults under 50 years, and these differences in the humoral response are primarily observed in older patients.

Our study ascertained the presence of neutralizing antibodies, employing the cPass™ SARS-CoV-2 Neutralization Antibody Detection Kit, which had demonstrated a high sensibility, specificity, and strong correlation with the gold standard [13]. The peak of neutralizing antibodies was observed during the first 21 days after the second dose, in which 95.27% of the participants displayed a positive presence of neutralizing antibodies. This proportion was superior to the study reported from Sri Lanka [19] but inferior to the data reported in phases 1 and 2 of the BBIBP-Corv-2 clinical trials [18].

Notably, we showed that the PI group had outstanding NAbs levels provoked by the BBIBP-CorV-2 booster effect in all the evaluation periods. Even after 180 days from the second dose, their values were higher than those observed at the peak of neutralizing antibodies (21 days after the second dose) from the NPI group, suggesting that vaccination after a natural infection process might confer more robust longer protection. This particularity has also been described in the BNT162b2 vaccine. A single dose of this vaccine on PI individuals provoked a similar humoral response to the response evaluated on NPI individuals with two doses [23,24].

We observed that the humoral immunity decreased progressively along the study period. This was particularity noticeable on NAbs, where only 47.32% of the HCW NPI showed positive results 180 days after the second dose. These findings were similar to those reported by Yihao et al., where only 28% of the participants immunized with BBIBP-CorV-2 showed, after 180 days, positive neutralizing antibodies [25]. The NAbs decrease phenomenon, over time, has also been reported in other types of vaccines, such as CoronaVac [26], BNT162b2 [27,28], mRNA-1273 [29], Ad26.CoV2.S [30] and Gam-COVID-Vac [31]. Nonetheless, even with this decrease, a study found that 97.47% of participants vaccinated with BNT162b2 have shown NAbs until 180 days after, which represents 2.4 times more than observed with BBIBP-CorV-2 [32], as seen in previously infected BBIBP vaccinated HCWs. Similarly, another study that evaluated people immunized with mRNA-1273 reported that the neutralizing activity against most new variants of the virus was still intense on them, even after 180 days [33].

To adjust the spectrum of the antibodies measured induced by the BBIBP-CorV-2 vaccine, we employed the Elecsys^®^ anti-SARS-CoV-2 S assay, which only detects high-affinity antibodies and makes it possible to detect circulating NAbs. Even when it has not been designed or approved for this purpose, our finding showed a strong correlation between it and the cPass™ SARS-CoV-2 Neutralization Antibody Detection Kit, even superior to the one reported by Trougakos et al. [14]. A previous study also revealed that anti-S-RBD IgG values superior to 8.3, 44.6, and 334.2 U/mL could predict, with a sensibility greater than 90% and a specificity greater than 96%, a XPSI of the Ab neutralization cPass™ superior to 30%, 50%, and 75%, respectively. These findings could be the foundation to justify using the Elecsys^®^ anti-SARS-CoV-2 S as a strong predictor of the neutralized antibody levels, with the advantage that it provides faster results at a lower cost than other assays.

Finally, the findings in this study described in detail the humoral response after immunization with BBIBP-CorV-2 vaccination and are relevant due to the correlation between levels of NAbs and protection against COVID-19. It has been proved that the higher level of NAbs offers protection around 98%; meanwhile, in those whose levels are 1000 times lower, protection was reduced to 78% [10]. Likewise, a study in Israel established that the apparition of irruptive SARS-CoV-2 infections in HCW vaccinated with BNT162b2 was associated with lower peri-infection neutralizing antibody titers (2.7 times less) than those without infection [34].

It is relevant to mention that humoral immunity is not the only protective response against SARS-CoV-2. It has been observed that patients vaccinated with CoronaVac who responded with NAbs had a significantly higher level of anti-spike IgG and a tendency to generate more spike-specific memory B cells than non-responders. They also showed similar titers of spike-specific memory CD8 T cells and CD4 T cells compared with convalescent patients. Furthermore, this adaptive immunity has been associated with a reduction in disease severity [23,26].

Among the limitations of our study, we can point out that all the participants were HCW, most of them apparently in good health conditions (without comorbidities). For that reason, the results cannot be extrapolated to the general population. Also, the retrospective design based on a secondary study database does not allow us to know more details of the HCW, such as specific comorbidities, the severity of COVID-19 symptoms, and others. Additionally, we did not have control over the level of NAbs before vaccination since, during the first phase of the study, these tests were not available in our country, and these data could be relevant. The loss of participants during the follow-up, provoked by participants refraining from receiving a second dose or abandoning the laboratory controls follow-up, might have created a bias. The possible bias caused by the study’s measurement is not relevant because the instruments and assays used in this study were approved previously by regulatory entities.

## Figures and Tables

**Table 1:** Demographics characteristics and humoral response rates of the study population and comparison between previously infected and not previously infected.

Variable	Total (*n* = 252)	Previously Infected (*n* = 121)	Not Previously Infected (*n* = 131)	*p*-Value
Age, years * (IQR)	35 (29–45)	35 (29.5–45)	35.5 (29–46)	0.914 ^a^
Sex (%)				0.269 ^b^
- Female	203 (80.55)	94 (46.31)	109 (53.69)	
- Male	49 (19.45)	27 (55.10)	22 (44.9)	
Laboral Area (%)				0.203 ^b^
- Phlebotomy	78 (30.95)	42 (53.85)	36 (46.15)	
- Customer service	52 (20.63)	24 (46.15)	28 (53.85)	
- Maintenance service	28 (11.11)	17 (60.71)	11 (39.29)	
- Analytic Process	35 (13.90)	12 (34.29)	23 (65.71)	
- Administrative	59 (23.41)	26 (44.07)	33 (55.93)	
Working mode (%)				0.911 ^b^
- Remote	35 (14.17)	17 (48.57)	18 (51.43)	
- Presence	198 (78.35)	96 (48.48)	102 (51.52)	
- Both	19 (7.54)	9 (47.37)	10 (52.63)	
Days since infection * (IQR)		231.5 (173–277)		
Humoral response rates **				
21 days after the first dose (%)				
- SARS-CoV-2 S1 IgM/IgG	162 (71.68)	97 (95.09)	65 (52.41)	<0.001 ^b^
- Anti-SARS-CoV-2-N	117 (49.16)	110 (98.21)	7 (5.56)	<0.001 ^c^
- Seroconversion	176 (73.95)	111 (99.10)	65 (51.59)	<0.001 ^c^
21 days after the second dose (%)				
- SARS-CoV-2-S1 IgM/IgG	241 (95.63)	121 (100)	120 (91.60)	<0.001 ^b^
- Anti-SARS-CoV-2 N	211 (88.66)	121 (100)	90 (68.70)	<0.001 ^c^
- Elecsys® anti-SARS-CoV-2 S	248 (98.41)	121 (100)	127 (96.94)	0.071 ^c^
- Ab neutralization cPass™	222 (95.28)	106 (99.06)	116 (92.06)	0.013 ^c^
- Seroconversion	241 (95.63)	121 (100)	120 (91.60)	<0.001 ^c^
90 days after the second dose (%)				
- Elecsys® anti-SARS-CoV-2 S	224 (99.56)	109 (100)	115 (99.14)	0.516 ^c^
- Ab neutralization cPass™	196 (87.11)	109 (100)	87 (75)	<0.001 ^c^
180 days after second dose (%)				
- Elecsys® anti-SARS-CoV-2 S	224 (99.56)	113 (100)	111 (99.11)	0.498 ^c^
- Ab neutralization cPass™	164 (72.89)	111 (98.23)	53 (47.32)	<0.001 ^c^

* Median and interquartile range; ^a^ U-Mann–Whitney; ^b^ χ2; ^c^ Fisher’s exact. IQR: Interquartile range. ** The distribution of the humoral response percentages was calculated based on the population described in Figure 1.

**Table 2:** Humoral response rates by SARS-CoV-2 specific antibody levels of the study population and comparison between previously infected and previously uninfected.

Variable	Total (*n* = 268)	Previously Infected (*n* = 121)	Not Previously Infected (*n* = 147)	*p*-Value
IgM/IgG SARS-CoV-2 S1 (UI/mL) *				
- Baseline	3.3 (0.4–23.5)	23.8 (10.3–85.3)	0.95 (0.4–1.75)	<0.001 ^a^
- 21 days after first dose	21.7 (5.8–64.1)	65.45 (44.6–102)	8.4 (3.7–17.2)	<0.001 ^a^
- 21 days after second dose	66.1 (35.45–105.7)	82.5 (57.9–110.9)	49.5 (25.7–89.8)	<0.001 ^a^
IgM/IgG/IgA anti-SARS-CoV-2 N (S/Co) *(IQR)				
- Baseline	0.1 (0.1–24.65)	25.9 (9.1–68.5)	0.1 (0.1–0.1)	<0.001 ^a^
- 21 days after first dose	0.7 (0.1–128.1)	135.6 (85.8–190.3)	0.1 (0.1–0.2)	<0.001 ^a^
- 21 days after second dose	37.4 (2.75–155.35)	159.7 (93.8–211.4)	2.9 (0.5–7.8)	<0.001 ^a^
Ab neutralization cPass™ (%) * (IQR)				
- 21 days after second dose	88.1 (68–96.3)	96.4 (92.4–97.1)	73.5 (53.9–85.2)	<0.001 ^a^
- 90 days after second dose	72.4 (45.1–94.3)	94.3 (82.9–96.1)	45.6 (30.85–65.7)	<0.001 ^a^
- 180 days after second dose	63.6 (27.8–92.2)	88.5 (72.4–95.8)	28.15 (16.9–43.55)	<0.001 ^a^
Elecsys^®^ anti-SARS-CoV-2 S (UI/mL) * (IQR)				
- 21 days after second dose	293.45 (92.75–1275)	1288 (715.9–2334)	117.4 (48.7–222.8)	<0.001 ^a^
- 90 days after second dose	187.1 (49–737.4)	714.7 (414–1347)	49.95 (28.05–101.25)	<0.001 ^a^
- 180 days after second dose	296.2 (63.9–782.1)	558.2 (356.8–1135)	64.2 (29.4–146.5)	<0.001 ^a^

* Median and interquartile range, ^a^ U-Mann–Whitney, IQR: Interquartile range.

**Table 3:** Simple and multiple linear regression of the variables associated with titles of Ab neutralization cPass™ and Elecsys^®^ anti-SARS-CoV-2 S.

Variable	Crude Beta-Coefficient 95%	*p*-Value	Adjusted Beta-Coefficient 95%	*p*-Value
Ab Neutralization cPass				
Male sex	17.076 (6.325–27.827)	0.002	11.372 (−1.278–24.023)	0.078
Previously infected	45.211 (39.964–51.457)	<0.001	44.692 (38.487–50.897)	<0.001
Elecsys^®^ Anti-SARS-CoV-2 S				
Male sex	385.629 (134.249–637.008)	0.003	334.735 (97.651–571.820)	0.006
Previously infected	540.221 (351.556–728.886)	<0.001	519.961 (333.527–706.395)	<0.001

## Data Availability

The data analyzed in this manuscript, as well as its definitions, could be downloaded at: https://data.mendeley.com/datasets/mnjnh69jsg/2 (accessed on 8 March 2022).
